# Supplementary material for: Structural bases for aspartate recognition and polymerization efficiency of cyanobacterial cyanophycin synthetase
Source: Nat Commun. 2022 Aug 30;13:5097. doi: 10.1038/s41467-022-32834-8 (PMC9427784; doi:10.1038/s41467-022-32834-8)
Supplement: Supplementary file 1 — Reporting Summary [file 41467_2022_32834_MOESM1_ESM.pdf]

nature portfolio

Corresponding author(s): Masaru Tanokura, Toshiya Senda

Last updated by author(s): Jul 27, 2022

Reporting Summary

Nature Portfolio wishes to improve the reproducibility of the work that we publish. This form provides structure for consistency and transparency in reporting. For further information on Nature Portfolio policies, see our Editorial Policies and the Editorial Policy Checklist.

Statistics

For all statistical analyses, confirm that the following items are present in the figure legend, table legend, main text, or Methods section.

n/a	Confirmed	
☐	☒	The exact sample size (*n*) for each experimental group/condition, given as a discrete number and unit of measurement
☐	☒	A statement on whether measurements were taken from distinct samples or whether the same sample was measured repeatedly
☒	☐	The statistical test(s) used AND whether they are one- or two-sided<br>*Only common tests should be described solely by name; describe more complex techniques in the Methods section.*
☒	☐	A description of all covariates tested
☐	☒	A description of any assumptions or corrections, such as tests of normality and adjustment for multiple comparisons
☐	☒	A full description of the statistical parameters including central tendency (e.g. means) or other basic estimates (e.g. regression coefficient) AND variation (e.g. standard deviation) or associated estimates of uncertainty (e.g. confidence intervals)
☒	☐	For null hypothesis testing, the test statistic (e.g. *F*, *t*, *r*) with confidence intervals, effect sizes, degrees of freedom and *P* value noted<br>*Give P values as exact values whenever suitable.*
☒	☐	For Bayesian analysis, information on the choice of priors and Markov chain Monte Carlo settings
☒	☐	For hierarchical and complex designs, identification of the appropriate level for tests and full reporting of outcomes
☒	☐	Estimates of effect sizes (e.g. Cohen's *d*, Pearson's *r*), indicating how they were calculated

Our web collection on statistics for biologists contains articles on many of the points above.

Software and code

Policy information about availability of computer code

Data collection	Cryo-EM data: EPU 1.10 and SerialEM 3.8.
Data analysis	EM data processing: RELION 3.1, MotionCor2 1.1.0, 1.3.0, 1.4.2, Gctf 1.18, CTFFIND 4.1, SPHIRE crYOLO 1.4.1, 1.6.1, and 3DFSC. Model building and refinement: UCSF Chimera 1.13.1, Coot 0.8.9.2, PHENIX 1.19.2, SWISS-MODEL, and AlphaFold2. Structure viewer: UCSF Chimera 1.13.1 and PyMol 2.4.0. Analysis of activity assay data: GraphPad Prism 9.40. Bioinformatics: Protein BLAST (BLAST+ 2.12.0), CLUSTAL W 2.1, ESPript 3.0, and WebLogo 2.8.2.

For manuscripts utilizing custom algorithms or software that are central to the research but not yet described in published literature, software must be made available to editors and reviewers. We strongly encourage code deposition in a community repository (e.g. GitHub). See the Nature Portfolio guidelines for submitting code & software for further information.

Data

Policy information about availability of data

All manuscripts must include a data availability statement. This statement should provide the following information, where applicable:

- Accession codes, unique identifiers, or web links for publicly available datasets
- A description of any restrictions on data availability
- For clinical datasets or third party data, please ensure that the statement adheres to our policy

Cryo-EM maps generated in this study have been deposited in the Electron Microscopy Data Bank (EMDB) under accession codes EMD-32381 [https://www.ebi.ac.uk/pdbe/entry/emdb/EMD-32381] for TeCphA1 in the apo state, EMD-32382 [https://www.ebi.ac.uk/pdbe/entry/emdb/EMD-32382] for TeCphA1 bound with ATPγS, and EMD-32383 [https://www.ebi.ac.uk/pdbe/entry/emdb/EMD-32383] (C1) and EMD-32384 [https://www.ebi.ac.uk/pdbe/entry/emdb/EMD-32384] (C2) for TeCphA1 bound with ATPγS, aspartate, and (β-Asp-Arg)4. The structural coordinates are available in the PDB under accession codes 7WAC

[http://doi.org/10.2210/pdb7WAC/pdb] for TeCphA1 in the apo state, 7WAD [http://doi.org/10.2210/pdb7WAD/pdb] for TeCphA1 bound with ATPγS, and 7WAE [http://doi.org/10.2210/pdb7WAE/pdb] (C1) and 7WAF [http://doi.org/10.2210/pdb7WAF/pdb] (C2) for TeCphA1 bound with ATPγS, aspartate, and (β-Asp-Arg)4. Enzymatic activity data generated in this study are provided in the Source Data file. The PDB coordinates used in this study are as follows: 7LGJ [http://doi.org/10.2210/pdb7LGJ/pdb] and 7LGQ [http://doi.org/10.2210/pdb7LGQ/pdb]. Source data are provided with this paper.

Field-specific reporting

Please select the one below that is the best fit for your research. If you are not sure, read the appropriate sections before making your selection.

☒ Life sciences ☐ Behavioural & social sciences ☐ Ecological, evolutionary & environmental sciences

For a reference copy of the document with all sections, see nature.com/documents/nr-reporting-summary-flat.pdf

Life sciences study design

All studies must disclose on these points even when the disclosure is negative.

Sample size	The final 3D reconstructions for three states of TeCphA1 were performed using 161,823, 49,842, and 198,918 particles from the cryo-EM datasets comprising 2,109, 1,730, and 3,843 micrographs, respectively (shown in Supplementary Figs. 2–4). Enzymatic activity experiments were performed in quadruplets (when sufficient material was available) or triplicates as independent measurements. No sample-size calculation was performed.
Data exclusions	Micrographs were excluded based on the motion correction and the CTF estimation, and the particles were excluded by the 2D and 3D classification in RELION (shown in Supplementary Figs. 2–4).
Replication	Enzymatic activity experiments were performed in either triplicates or quadruplets.
Randomization	Particle datasets randomly split in two halves were used during the 3D auto-refinement steps in RELION. Resolution was assessed by the Fourier shell correlation between the half-maps at the 0.143 threshold criteria.
Blinding	Investigators were not blinded to group allocation during data collection and/or analysis because no group allocation involved in this study.

Reporting for specific materials, systems and methods

We require information from authors about some types of materials, experimental systems and methods used in many studies. Here, indicate whether each material, system or method listed is relevant to your study. If you are not sure if a list item applies to your research, read the appropriate section before selecting a response.

Materials & experimental systems

n/a	Involved in the study	
☒	☐	Antibodies
☒	☐	Eukaryotic cell lines
☒	☐	Palaeontology and archaeology
☒	☐	Animals and other organisms
☒	☐	Human research participants
☒	☐	Clinical data
☒	☐	Dual use research of concern

Methods

n/a	Involved in the study	
☒	☐	ChIP-seq
☒	☐	Flow cytometry
☒	☐	MRI-based neuroimaging
