# Supplementary material for: Structural bases for aspartate recognition and polymerization efficiency of cyanobacterial cyanophycin synthetase
Source: Nat Commun. 2022 Aug 30;13:5097. doi: 10.1038/s41467-022-32834-8 (PMC9427784; doi:10.1038/s41467-022-32834-8)
Supplement: Supplementary file 2 — Supplementary Info File #1 [file 41467_2022_32834_MOESM2_ESM.pdf]

Supplementary Information

Structural bases for aspartate recognition and polymerization efficiency of cyanobacterial cyanophycin synthetase

Takuya Miyakawa[1,§,¥], Jian Yang[1,2,3,§], Masato Kawasaki[4,5,§], Naruhiko Adachi[4,§], Ayumu Fujii[1], Yumiko Miyauchi[1], Tomonari Muramatsu[1], Toshio Moriya[4], Toshiya Senda[4,5,6]* & Masaru Tanokura[1]*

[1]Department of Applied Biological Chemistry, Graduate School of Agricultural and Life Sciences, The University of Tokyo, 1-1-1 Yayoi, Bunkyo-ku, Tokyo 113-8657, Japan.

[2]CAS Key Laboratory of Tropical Marine Bio-resources and Ecology, Guangdong Key Laboratory of Marine Materia Medica, South China Sea Institute of Oceanology, Chinese Academy of Sciences, Guangzhou, China.

[3]Southern Marine Science and Engineering Guangdong Laboratory (Guangzhou), Guangzhou, China.

[4]Structural Biology Research Center, Photon Factory, Institute of Materials Structure Science, High Energy Accelerator Research Organization (KEK), 1-1 Oho, Tsukuba, Ibaraki 305-0801, Japan

[5]Department of Materials Structure Science, School of High Energy Accelerator Science, The Graduate University of Advanced Studies (Soken-dai), 1-1 Oho, Tsukuba, Ibaraki 305-0801, Japan

[6]Faculty of Pure and Applied Sciences, University of Tsukuba, 1-1-1 Tennodai, Ibaraki 305-8571, Japan

[§] These authors contributed equally.

[¥] Present address: Division of Integrated Life Science, Graduate School of Biostudies, Kyoto University, Kitashirakawa-oiwakecho, Sakyo-ku, Kyoto, 606-8502, Japan

*Corresponding author e-mails: amtanok@mail.ecc.u-tokyo.ac.jp (M.T.); toshiya.senda@kek.jp (T.S.)

Supplementary Table 1 Cryo-EM data collection, refinement and validation statistics

	*Te*CphA1 (EMD-32381) (PDB 7WAC)	*Te*CphA1 + ATPγS (EMD-32382) (PDB 7WAD)	*Te*CphA1 + ATPγS + (β-Asp-Arg)$_4$ + aspartate (EMD-32383, 32384) (PDB 7WAE, 7WAF)	
**Data collection and processing**				
Magnification	120,000×	120,000×	105,000×	
Voltage (kV)	200	200	300	
Electron exposure (e Å^{-2})	50	50	49	
Defocus range (μm)	–1.0 to –2.5	–1.0 to –2.5	–0.84 to –1.8	
Pixel size (Å)	0.88	0.88	0.83	
Initial particle images (no.)	556,730	927,603	1,371,057	
Final particle images (no.)	161,823	49,842	198,918	
Symmetry imposed	D_2	D_2	C_1	C_2
Map resolution (Å)	2.91	2.96	2.64	2.52
FSC threshold	0.143	0.143	0.143	0.143
Map resolution range (Å)	2.8–4.0	2.8–4.4	2.5–5.3	2.4–4.8
**Refinement**				
Model resolution (Å)	2.91	2.96	2.64	2.52
FSC threshold	0.143	0.143	0.143	0.143
Map sharpening *B* factor (Å^2)	Not used	Not used	Not used	Not used
Model composition				
Nonhydrogen atoms	22,016	22,268	24,851	24,842
Protein residues	22,016	22,016	24,386	24,386
Ligands	–	252	465	456
B-factors (Å^2)				
Protein	142.1	131.1	92.22	90.54
Ligand	–	124.8	81.87	82.26
R.m.s. deviations				
Bond lengths (Å)	0.002	0.003	0.003	0.003
Bond angles (°)	0.517	0.584	0.522	0.537
Validation				
MolProbity score	1.45	1.54	1.62	1.64
Clashscore	5.30	6.00	7.66	7.24
Poor rotamers (%)	0	0	0.04	0
Ramachandran plot (%)				
Favored region	97.09	96.71	96.81	96.40
Allowed region	2.91	3.29	3.19	3.60
Outliers	0.00	0.00	0.00	0.00

Supplementary Table 2 Primers for the generation of *Te*CphA1 mutants

Mutants	Primers
E215A	F: CACTTGCTTGTGACAAAGAAGGCAC<br>R: CTACACCAAGAATTCCGGTTTGGTTACTTAG
H267A	F: GGGTCGTGGCATTACCATAGATGTT<br>R: GCATTTCCGTCTAGAGGTTTAATGACAATAGGATAG
R323A	F: CAGTTCCAGCCCATGTGGTG<br>R: CTTCAGCTACAGCTACAACTTTGCC
N394A	F: GCTAAGCACTGGTGGTATAGCAGTAG<br>R: GCAGCCGTTGCTCTTAAAAAGCAC
R458A	F: CGATGCATGTTGCTCCTAGTAGAGG<br>R: CGAAACCAGGGGCAGCATTAAC
K499A	F: GACAACTACTACACGACTACTGGCTCAT<br>R: GCTCCATTAGTGCCAGTAACAGACAATATTGG
F692L	F: AATGATTGCTAATGCTCTGGCAGC<br>R: AAAGGAGCTTTACCAGCCATTGT
A755G	F: CTTAGGTGGATTTGTCAGAAATTGGCC<br>R: CCTTCATAGCTAGCAGGATTATGAGCATAATC
ΔM_{lid}	F: CTCGAGCACCACCACCACC<br>R: AGCCACAAAAGTTGACAAACCTTTCC

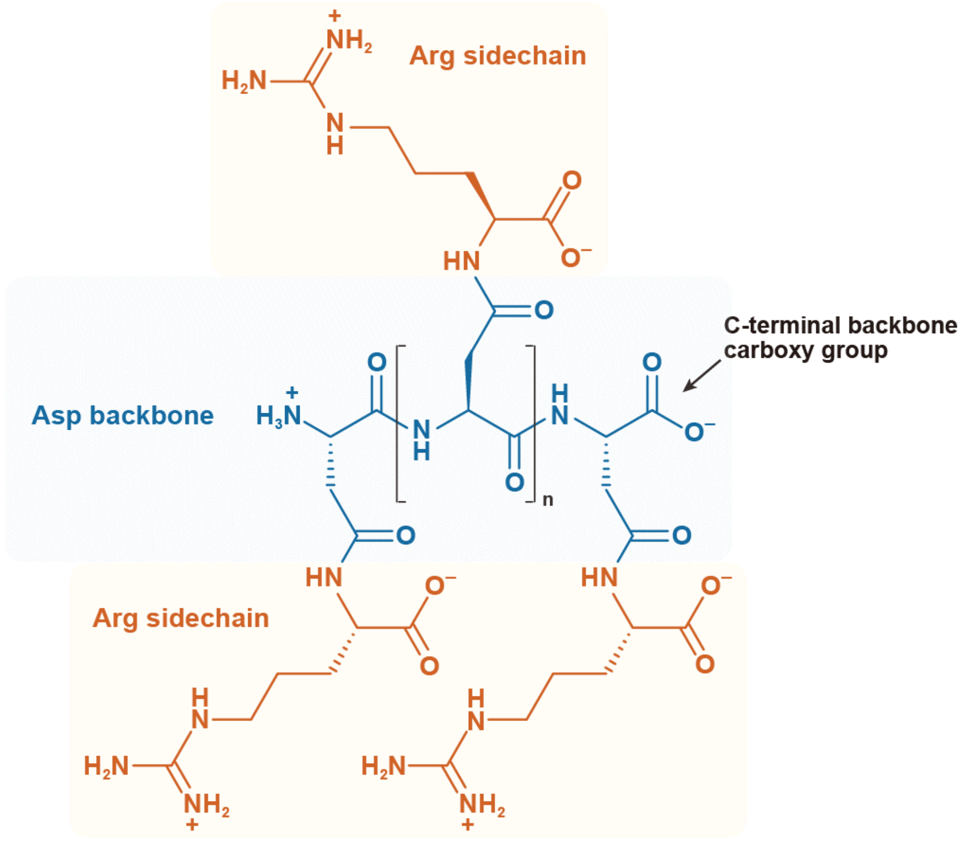

Supplementary Fig. 1 Chemical structure of cyanophycin. The backbone is composed of aspartate residues (Asp) linked by peptide bonds, and it is elongated by the condensation of Asp to the C-terminal backbone carboxy group. The arginine residue (Arg) is linked to the β-carboxy group of Asp as a sidechain by a peptide bond. Repeating unit is a β-Asp-Arg dipeptide.

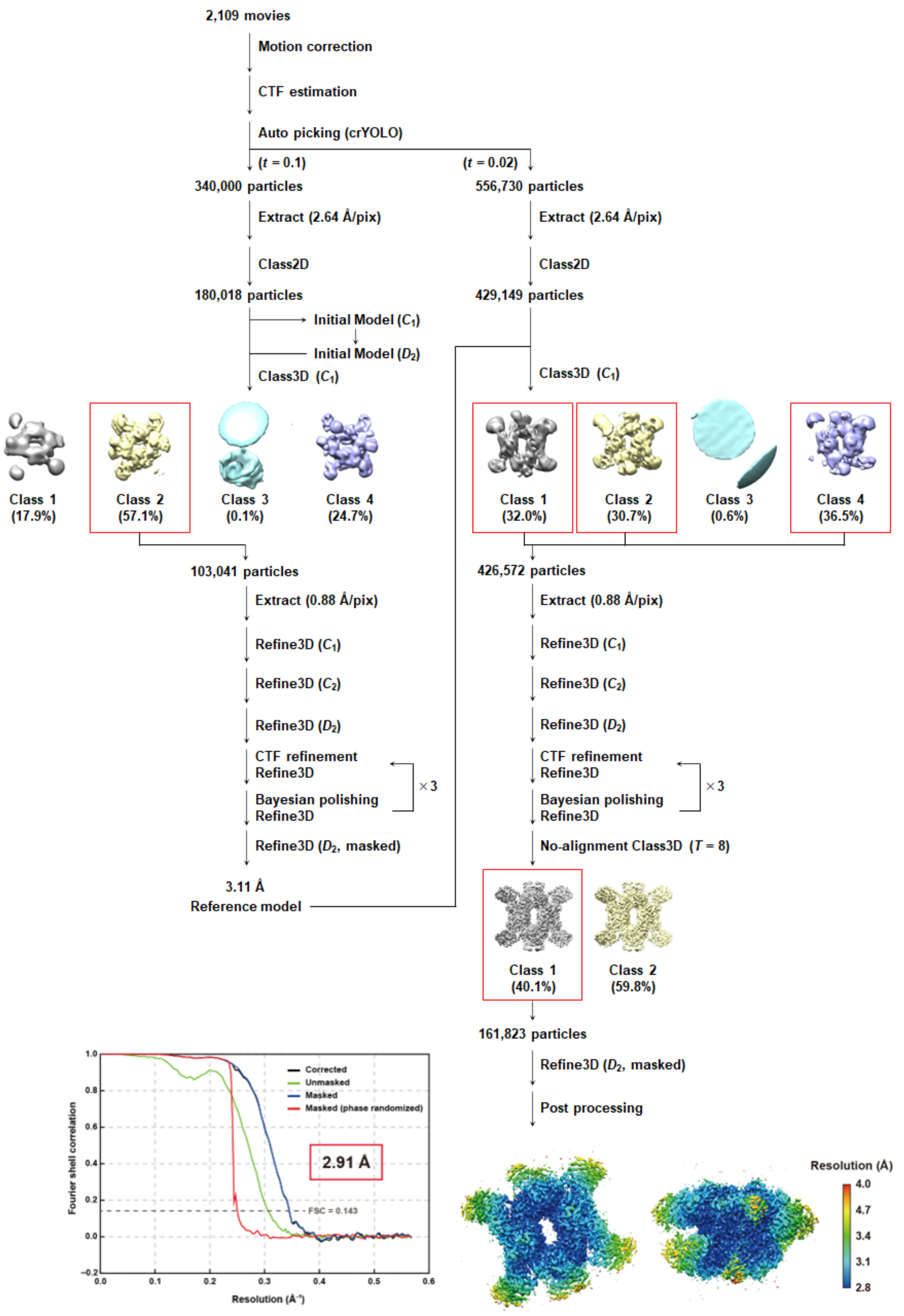

Supplementary Fig. 2 Cryo-EM analysis procedure of *Te*CphA1 in the apo state. Flow chart depicting the single particle analysis of *Te*CphA1 in the apo state. CTF, contrast transfer function; *t*, selection threshold; and *T*, regularization parameter. The Fourier shell correlation (FSC) curve shows a 2.91 Å global resolution with the gold standard criteria (FSC = 0.143). The local resolution is indicated on the cryo-EM map.

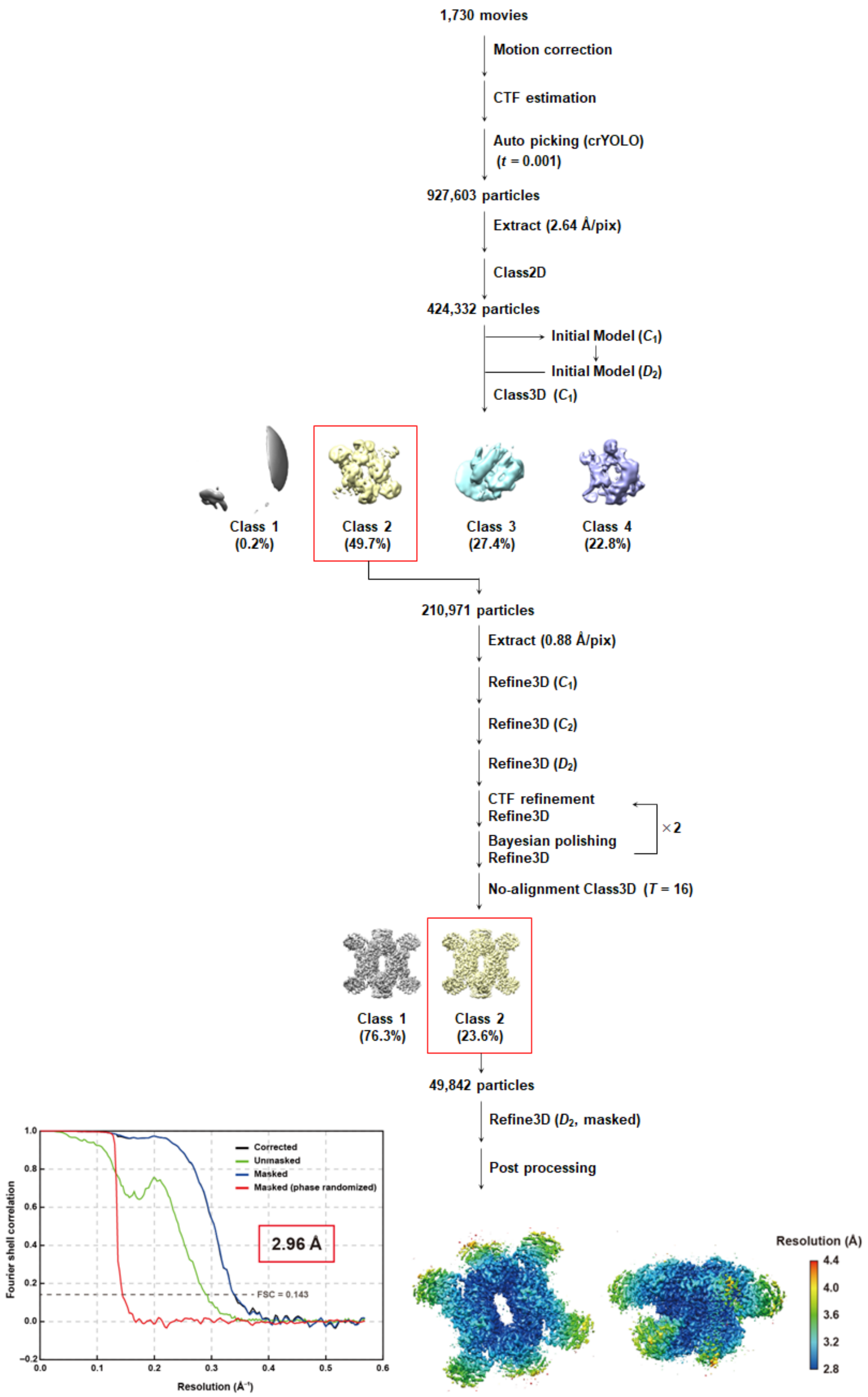

Supplementary Fig. 3 Cryo-EM analysis procedure of *Te*CphA1 in the ATPγS-bound state. Flow chart depicting the single particle analysis of *Te*CphA1 in the ATPγS-bound state. CTF, contrast transfer function; *t*, selection threshold; and *T*, regularization parameter. The Fourier shell correlation (FSC) curve shows a 2.96 Å global resolution with the gold standard criteria (FSC = 0.143). The local resolution is indicated on the cryo-EM map.

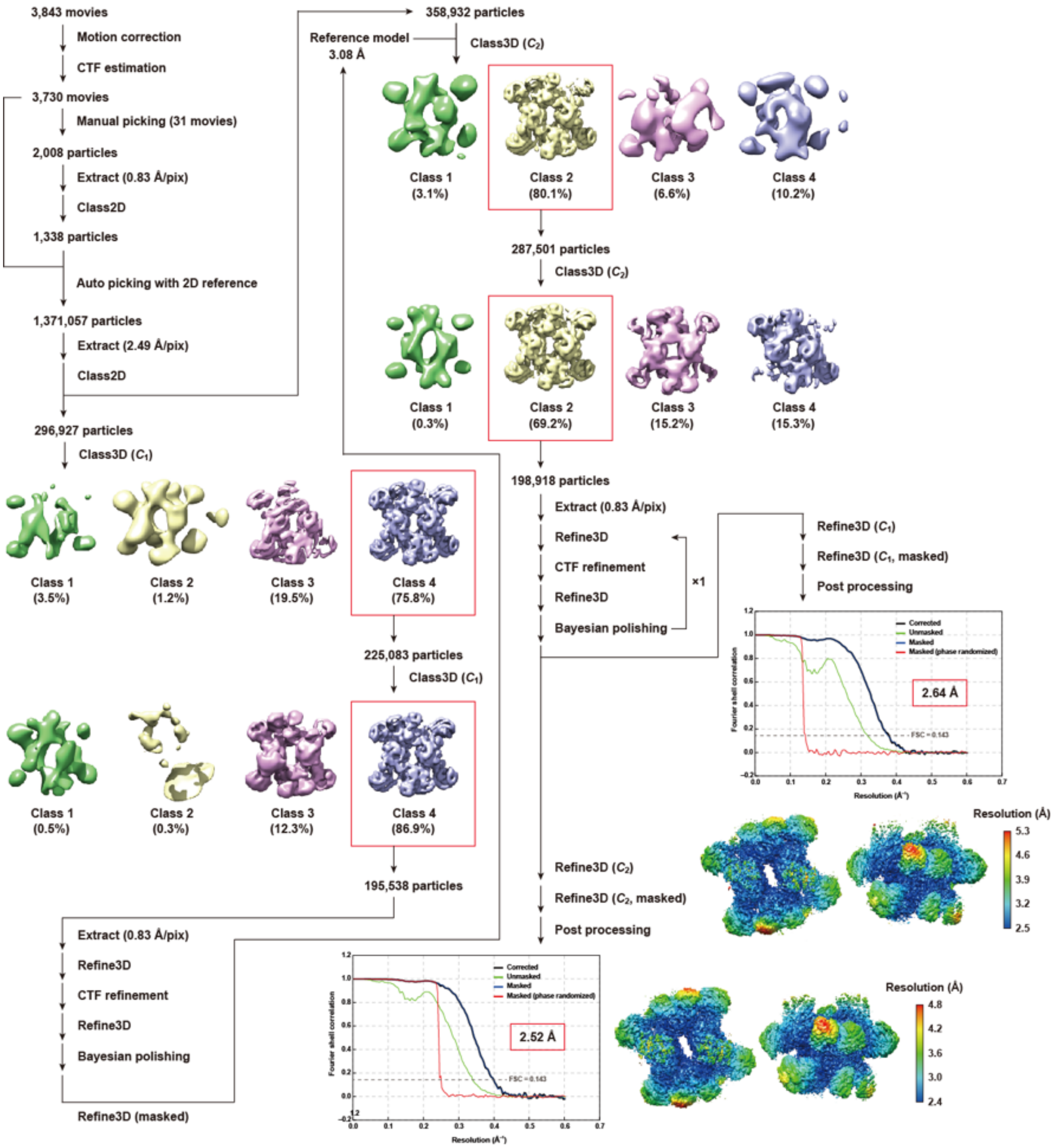

Supplementary Fig. 4 Cryo-EM analysis procedure of *Te*CphA1 in the substrate-bound state. Flow chart depicting the single particle analysis of *Te*CphA1 in the substrate-bound state. CTF means contrast transfer function. The Fourier shell correlation (FSC) curve shows 2.64 and 2.52 Å global resolutions with the gold standard criteria (FSC = 0.143) for the C_1 and C_2 symmetry maps, respectively. The local resolution is indicated on each cryo-EM map.

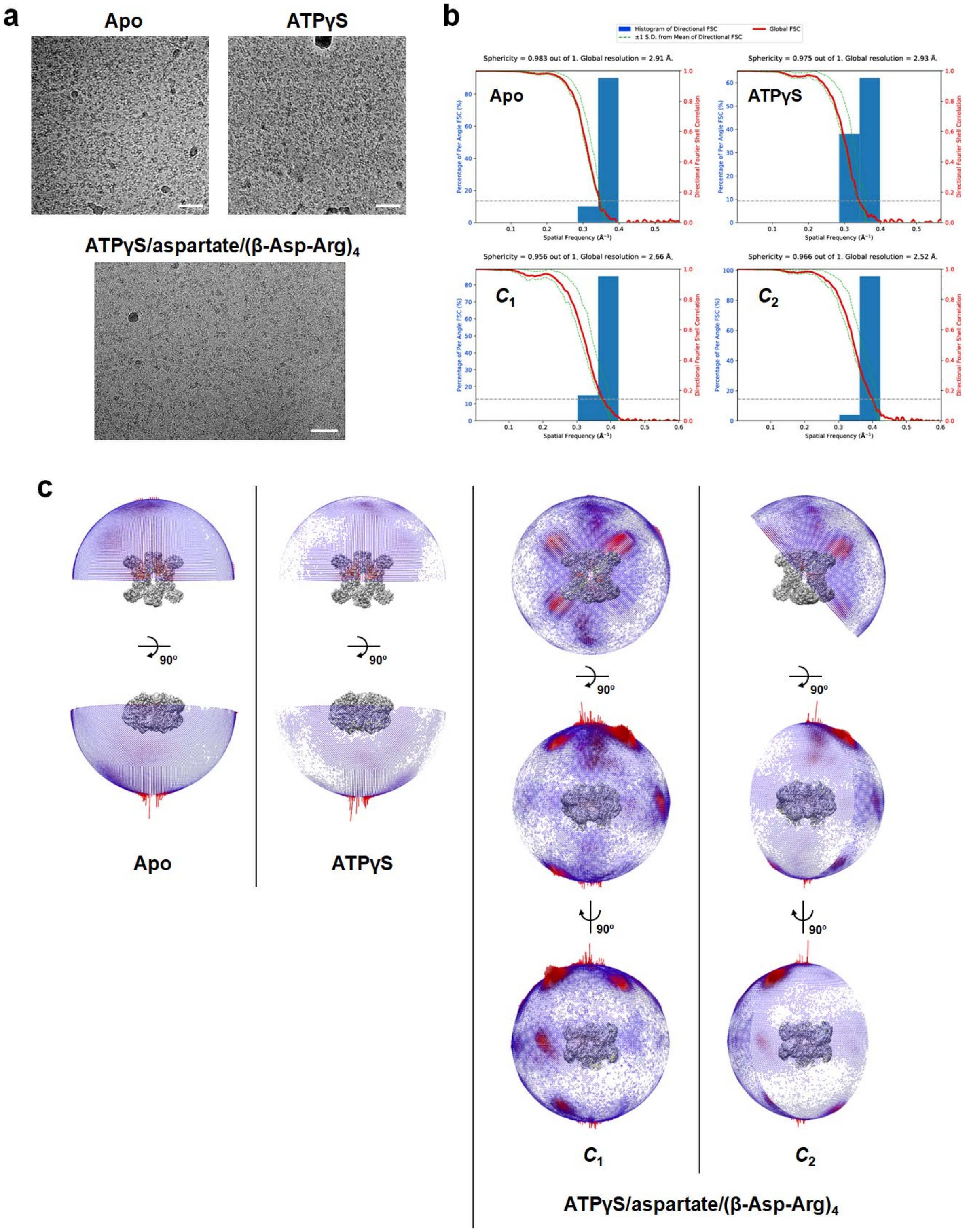

Supplementary Fig. 5 Cryo-EM micrographs and evaluation of the distribution of particle orientations, related to Supplementary Figs. 2–4. a Representative micrograph of each state of *Te*CphA1. Each experiment was repeated twice independently with similar results. Scale bar, 50 nm. **b** Directional Fourier shell correlation (FSC) plots calculated on the 3DFSC server[38] for

the reconstructions. The sphericity indicated above each plot is the degree of resolution anisotropy present in the associated reconstruction, and a histogram of a randomly selected subset of directional resolution values is also shown. **c** Angular distribution of cryo-EM particles. Blue, a small number of particles; and red, a large number of particles.

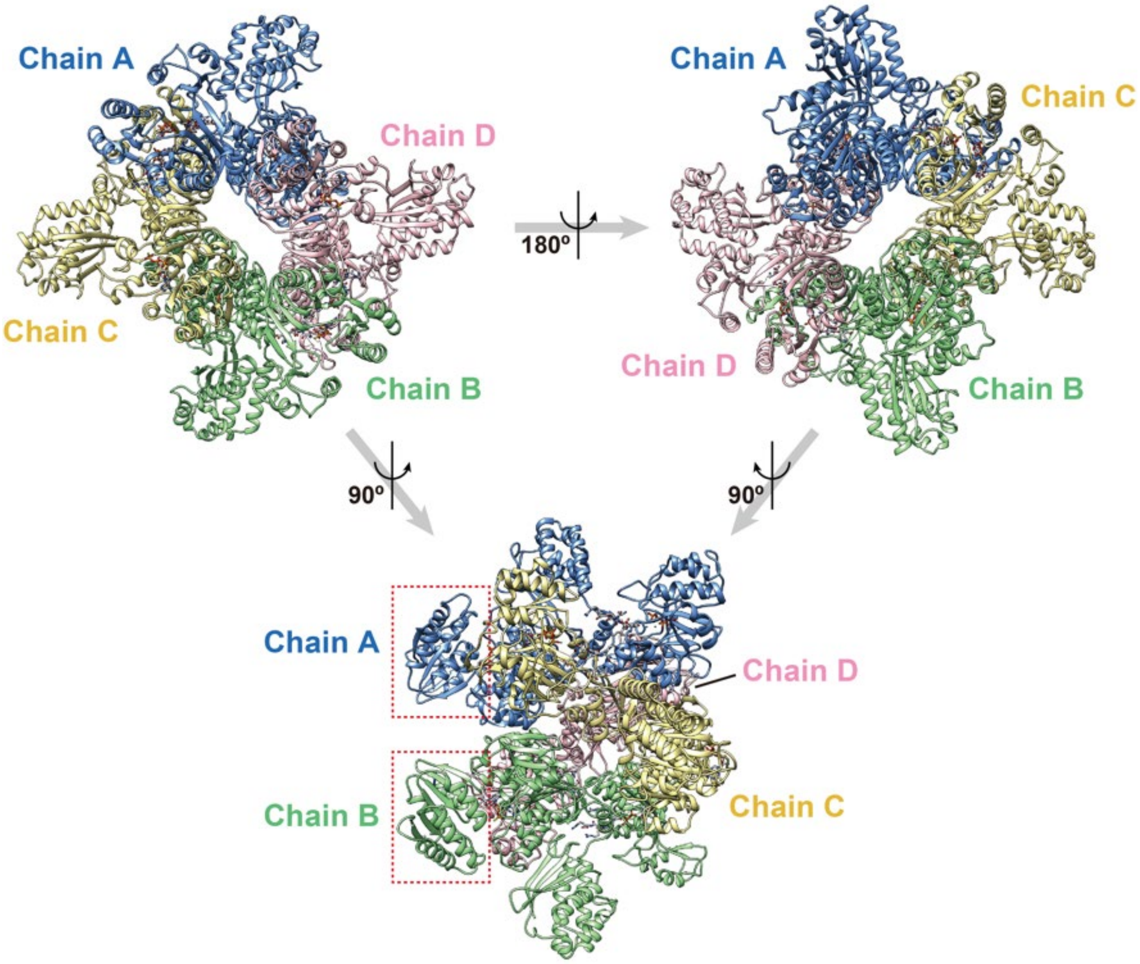

Supplementary Fig. 6 Tetrameric assembly of *Te*CphA1. Ribbon diagram of the tetrameric *Te*CphA structure in the substrate-bound state. The four protomers in the tetramer are shown in different colors. The red dashed box indicates the M_{lid} (residues 724–876) of chains A and B, and no M_{lid} model was built in chains C and D because of the lack of observations on the cryo-EM map.

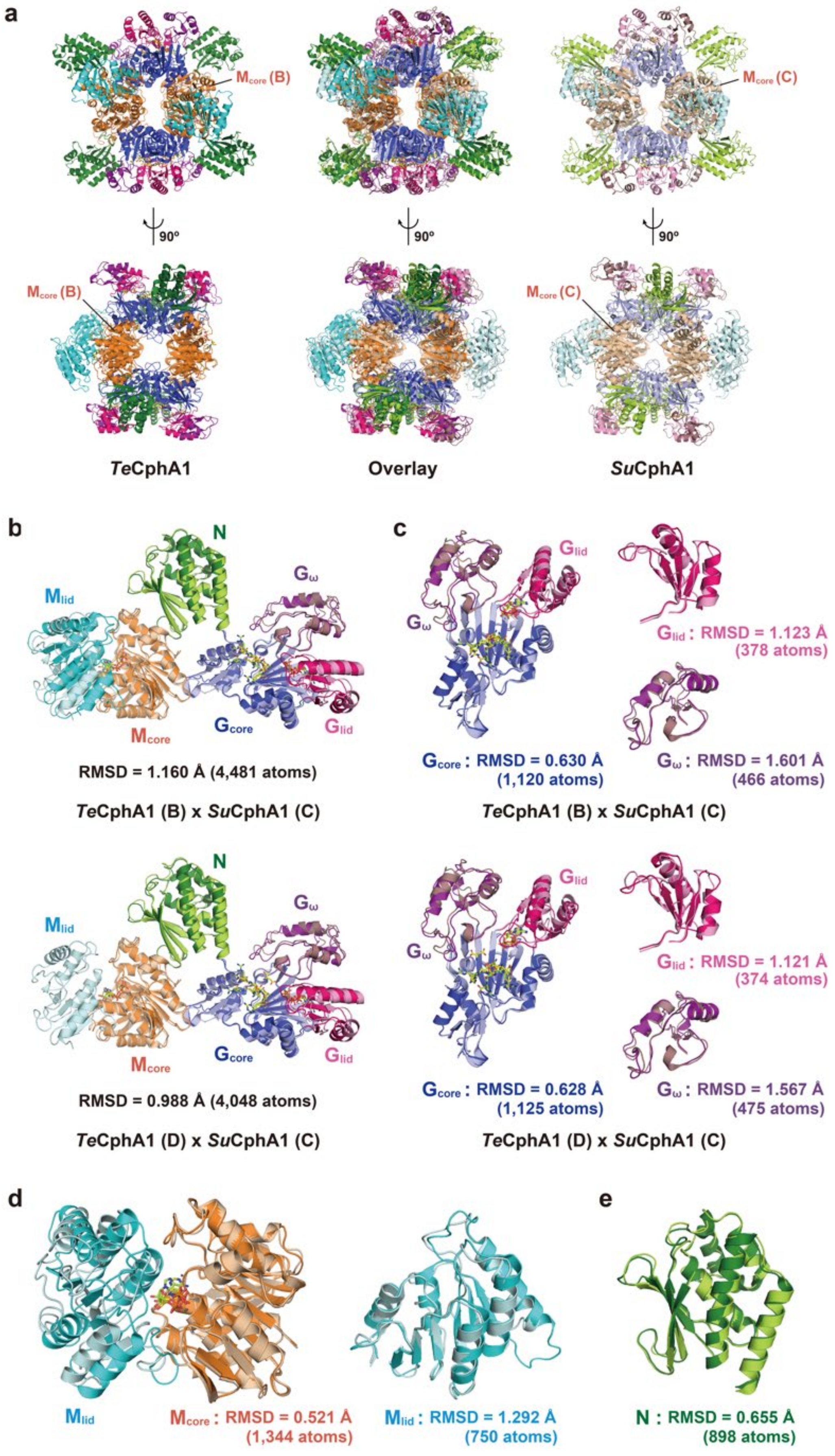
a
Mcore (B)
Mcore (C)
90º
90º
90º
Mcore (B)
Mcore (C)
TeCphA1
Overlay
SuCphA1
b
N
Mlid
Gω
Mcore
Gcore
Glid
RMSD = 1.160 Å (4,481 atoms)
TeCphA1 (B) x SuCphA1 (C)
c
Glid
Gω
Glid : RMSD = 1.123 Å
(378 atoms)
Gcore : RMSD = 0.630 Å
(1,120 atoms)
Gω : RMSD = 1.601 Å
(466 atoms)
TeCphA1 (B) x SuCphA1 (C)
N
Mlid
Gω
Mcore
Gcore
Glid
RMSD = 0.988 Å (4,048 atoms)
TeCphA1 (D) x SuCphA1 (C)
Glid
Gω
Glid : RMSD = 1.121 Å
(374 atoms)
Gcore : RMSD = 0.628 Å
(1,125 atoms)
Gω : RMSD = 1.567 Å
(475 atoms)
TeCphA1 (D) x SuCphA1 (C)
d
Mlid
Mcore : RMSD = 0.521 Å
(1,344 atoms)
Mlid : RMSD = 1.292 Å
(750 atoms)
e
N : RMSD = 0.655 Å
(898 atoms)

Supplementary Fig. 7 Structure comparison of *Te*CphA1 with *Su*CphA1. The *Te*CphA1 structure in the substrate-bound state (processed with C_1 symmetry) was superimposed on the *Su*CphA1 structure with a cyanophycin analog (β-Asp-Arg)$_8$-NH_2 on the G domain (PDB 7LGJ [http://doi.org/10.2210/pdb7LGJ/pdb]) using PyMol, and the structural similarity was calculated as a root-mean-square deviation (RMSD). **a** Comparison of the tetrameric assembly of *Te*CphA1 and *Su*CphA1. *Te*CphA1 M_{core} of chain B is superposed with *Su*CphA1 M_{core} of chain C. The color coordination of each protomer is consistent with that in panel **b**. **b** Comparison of two different *Te*CphA1 protomers (chains B and D) and the *Su*CphA1 protomer (chain C). The models of M_{lid} and the substrate aspartate are visible in chains B and D, respectively. The residues 1–876 of *Te*CphA1 and *Su*CphA1 correspond to the N, G and M domains. The sequence identity (similarity) of this region is 69.6% (83.2%). **c** Comparison of each module of the G domain of *Te*CphA1 and *Su*CphA1. The sequence identity (similarity) is 76.6% (86.8%) for G_{core}, 69.0% (85.9%) for G_{lid}, and 65.3% (86.7%) for G_{ω}. **d** Comparison of each module of the M domain of *Te*CphA1 (chain B) and *Su*CphA1 (chain C). The sequence identity (similarity) is 71.8% (85.0%) for M_{core} and 60.1% (73.2%) for M_{lid}. **e** Comparison of the N domain of *Te*CphA1 (chain B) and *Su*CphA1 (chain C). The sequence identity (similarity) is 72.0% (85.4%). The color coordination of the N domain and each module of *Te*CphA1 and *Su*CphA1 is the same among all the panels.

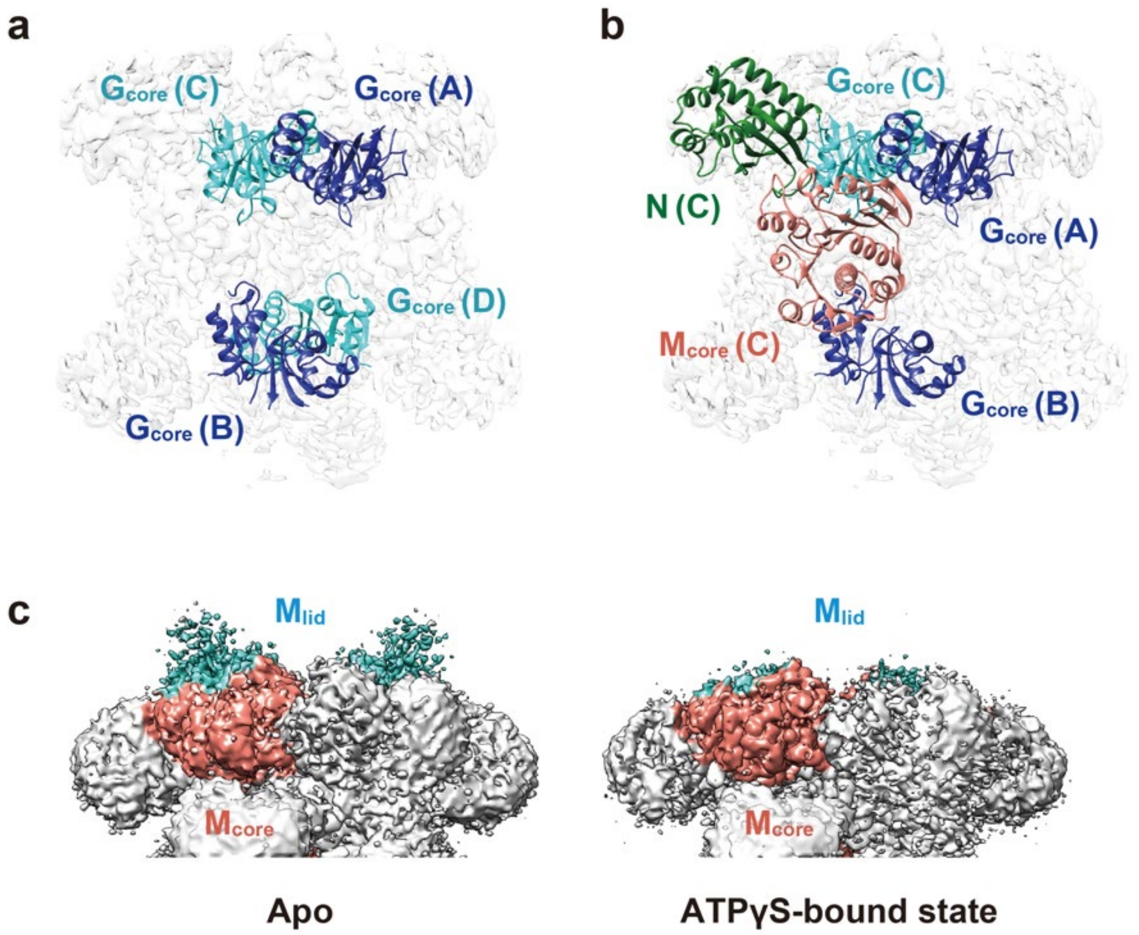

Supplementary Fig. 8 Stable and flexible modules in the *Te*CphA1 tetramer. a Dimer formation of G_{core}. Parentheses show the chain name of the *Te*CphA1 tetramer (A to D). **b** Contact of M_{core} with the intramolecular N domain and three G_{core}. Parentheses show the chain name of the *Te*CphA1 tetramer (A to D). **c** Comparison of the density map of M_{lid} in the apo and ATPγS-bound states.

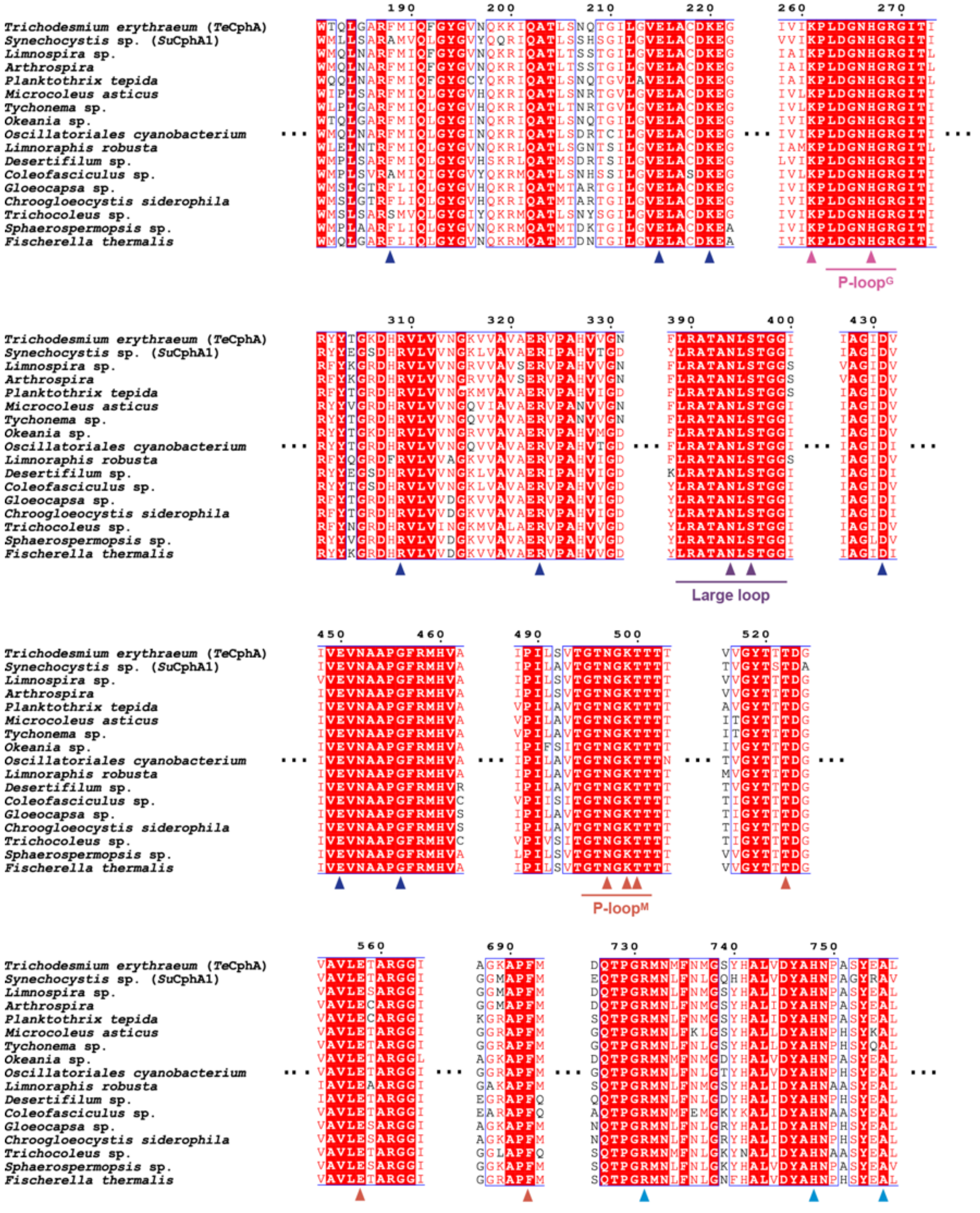

Supplementary Fig. 9 Amino acid sequence alignments of representative cyanobacterial CphA1. Triangles indicate the residues contributing to the interaction with Mg^{2+}, ATP, aspartate, and cyanophycin and/or catalytic activity of *Te*CphA1. The triangles colored in blue, pink, purple, salmon, and cyan show the residues on G_{core}, G_{lid}, G_{ω}, M_{core}, and M_{lid}, respectively.

National Center for Biotechnology Information (NCBI) accession numbers are as follows: *Trichodesmium erythraeum* (*Te*CphA1), MBS9770029.1; *Synechocystis* sp. (*Su*CphA1), WP_028947105.1; *Limnospira* sp., QNH58539.1; *Arthrospira* sp., WP_014275162.1; *Planktothrix tepida*, WP_072718573.1; *Microcoleus asticus*, WP_172192808.1; *Tychonema* sp., WP_194067582.1; *Okeania* sp., NET78835.1; *Oscillatoriales* cyanobacterium, OCR00164.1; *Limnoraphis robusta*, WP_046282003.1; *Desertifilum* sp., NES97515.1; *Coleofasciculus* sp., WP_190987081.1; *Gloeocapsa* sp., WP_015187686.1; *Chroogloeocystis siderophila*, WP_073550846.1; *Trichocoleus* sp., WP_190640035.1; *Sphaerospermopsis* sp., WP_187039540.1; and *Fischerella thermalis*, WP_102206793.1.

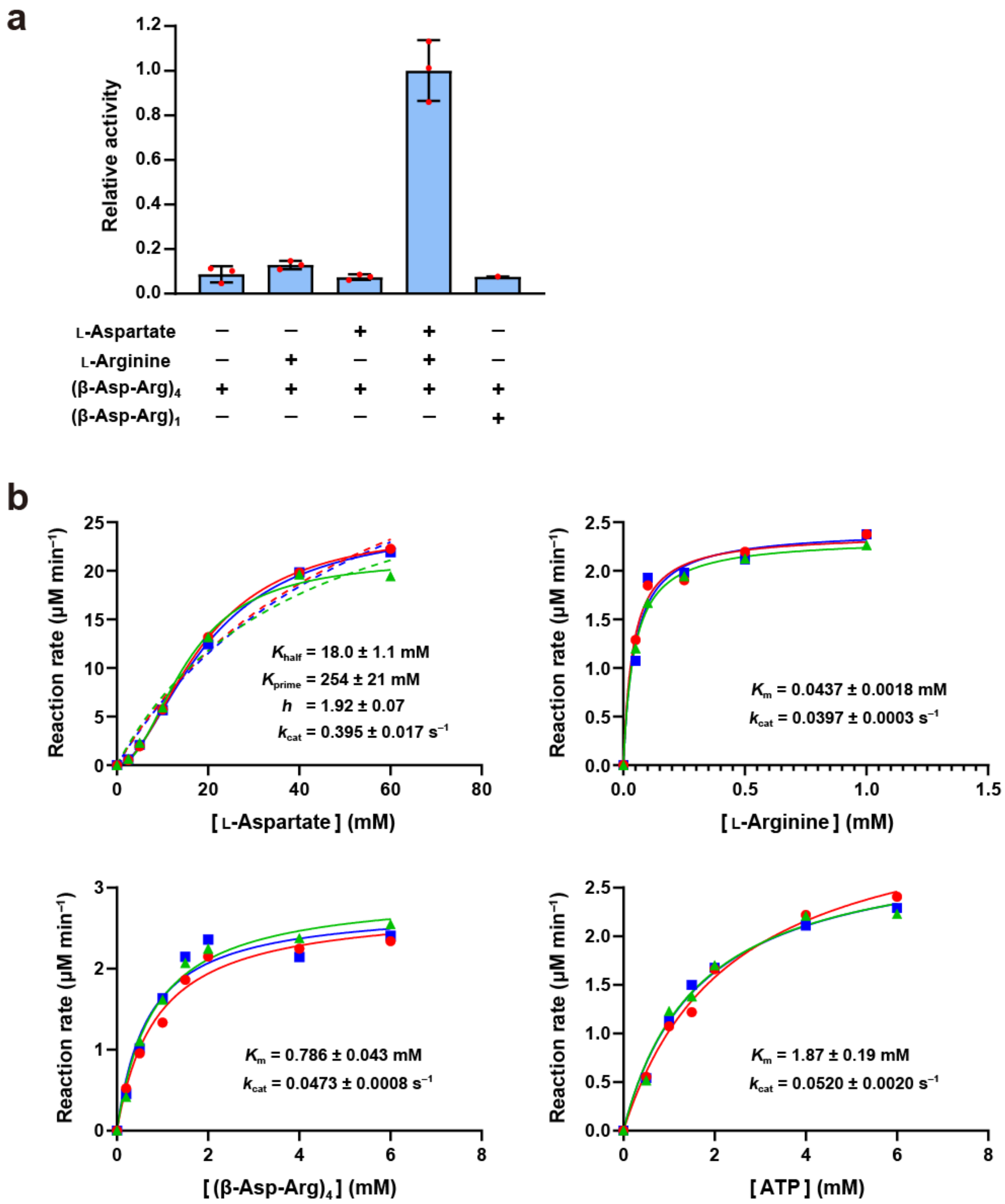

Supplementary Fig. 10 Basic characterization of the *Te*CphA1 reaction. a Relative activity of *Te*CphA1 with different substrate combinations. All reactions were performed in the presence of ATP. The *Te*CphA1 activity was calculated by subtracting the released phosphate concentration estimated using absorbance at 660 nm without *Te*CphA1 from that in the presence of the enzyme. The data are presented as mean ± standard error of the mean (SEM) (n = 3 independent experiments). **b** Steady-state kinetics of *Te*CphA1. The activity of *Te*CphA1 (1 μM as a tetramer) was measured using a released phosphate concentration in the presence of several concentrations of each substrate. The data for L-arginine, (β-Asp-Arg)$_4$, and ATP were

fit with a Michaelis-Menten curve. The curve fitting for L-aspartate was performed using an allosteric sigmoidal equation. Dashed lines are fitting curves using a Michaelis-Menten equation. Three data sets and the fitting curves are shown in different colors (red, blue, and green). The K_{m}, K_{half}, K_{prime}, h, and k_{cat} values are presented as the mean values ± SEM (n = 3 independent experiments).

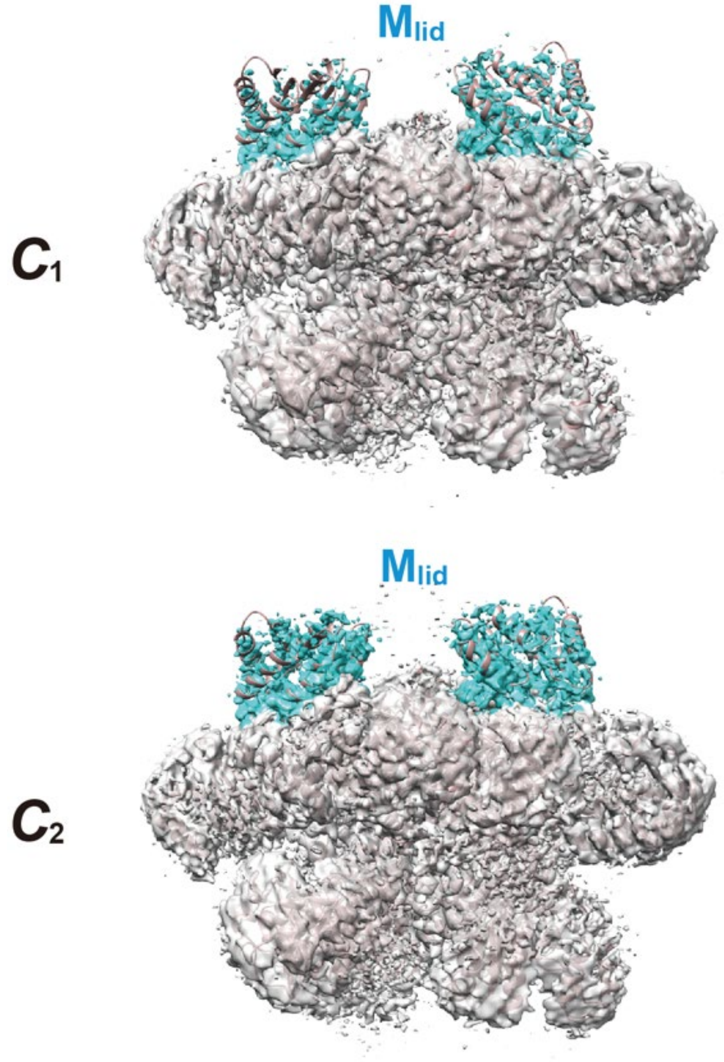

Supplementary Fig. 11 Comparison of the density maps of M_{lid} processed with C_1 and C_2 symmetry.

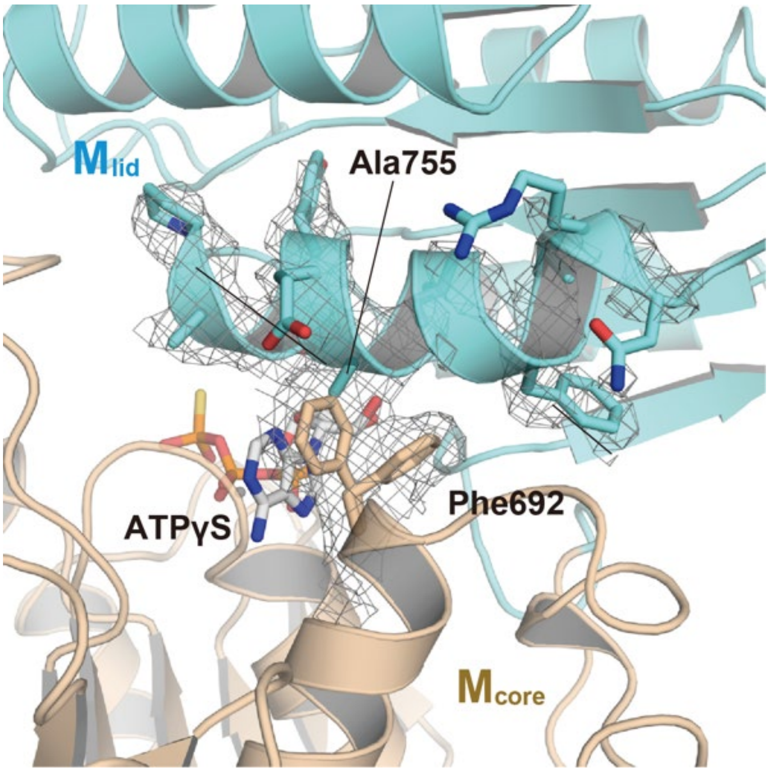

Supplementary Fig. 12 Two different orientations of Phe692 in the M_{lid}-bound state of *Te*CphA1. The cryo-EM map carved 2 Å around Phe692 and residues 755–762 on the M_{lid} module at contour level 4. Two conformations of Phe692 are depicted to fit the density map.

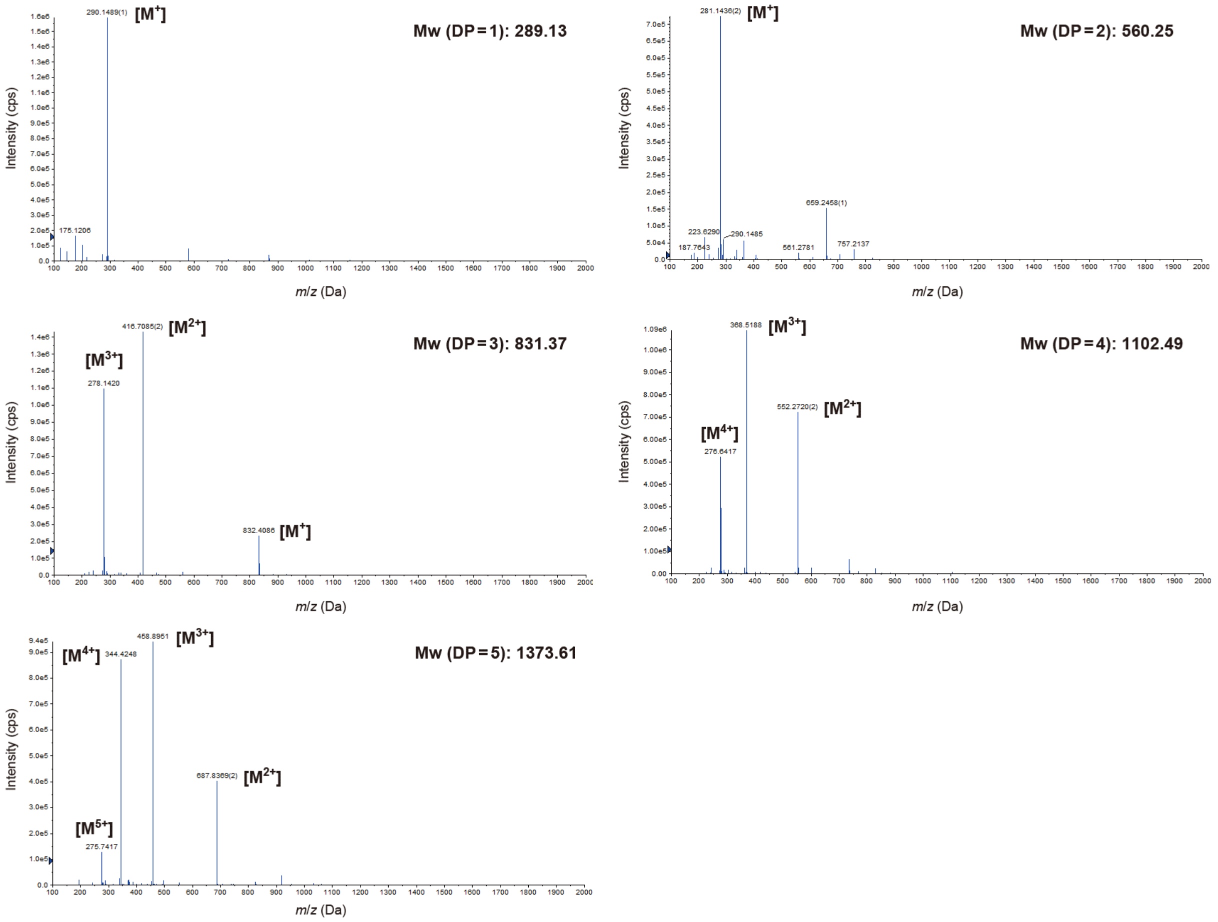

Supplementary Fig. 13 Time-of-flight mass spectrometry (TOF MS) spectra of synthetic cyanophycin peptides. Mw indicates the molecular weight of the β-Asp-Arg peptide with different degrees of polymerization (DP = 1–5).
